# Supplementary material for: How Bacteria Change after Exposure to Silver Nanoformulations: Analysis of the Genome and Outer Membrane Proteome
Source: Pathogens. 2021 Jun 29;10(7):817. doi: 10.3390/pathogens10070817 (PMC8308822; doi:10.3390/pathogens10070817)
Supplement: Supplementary file 1 [file pathogens-10-00817-s001.zip › supplementary Tables.pdf]

Supplementary materials

Table of content:

Table S1. Genes encoding outer membrane proteins selected with mutfunc and list of conservative and nonconservative mutations selected with BLOSUM62

Table S2. The other part of genes encoding proteins connected with outer membrane (OM), flagellum, fimbria, lipopolysaccharide (LPS), exopolysaccharide (EPS) selected with mutfunc and list of conservative mutations selected with BLOSUM62

Table S3. Regulatory genes selected with EcoCyc Database and list of conservative and non-conservative mutations selected with BLOSUM62

Table S1. Genes encoding outer membrane proteins selected with mutfunc and list of conservative and nonconservative mutations selected with BLOSUM62

Genes selected with mutfunc	Function	List of conservative mutations selected with BLOSUM62	List of non-conservative mutations selected with BLOSUM62
bamB	Outer membrane protein assembly factor BamB	Ser96Asn; Ser335Gly	Unselected
bamC	Outer membrane protein assembly factor BamC	Asp287Glu; Gln289His	Unselected
bcsC	Cellulose synthase operon protein C	Ala558Gly; Val65Ile; Ala775Thr	Pro110Ser
blc	Outer membrane lipoprotein Blc	Unselected	Gly84Glu
cirA	Colicin I receptor	Ile174Val	Unselected
cusC	Cation efflux system protein CusC	Thr81Ala; Asn117Asp; Thr118Ser	Unselected
fecA	Fe(3+) dicitrate transport protein FecA (Iron(III) dicitrate transport protein FecA)	Ser684Ala; Gly600Asp; Met693Ile; Val605Ile; Val350Leu; Ile56Phe; Leu651Phe;Ile397Val	Unselected
fepA	Ferrienterobactin receptor (Enterobactin outer-membrane receptor)	Thr356Ala	Unselected
fhuA	Ferrichrome outer membrane transporter/phage receptor (Ferric hydroxamate receptor) (Ferric hydroxamate uptake) (Ferrichrome-iron receptor)	Ser110Ala	Unselected
fhuE	FhuE receptor (Outer-membrane receptor for Fe(III)-coprogen, Fe(III)-ferrioxamine B and Fe(III)-rhodotrulic acid)	Ser61Asn	Unselected
fimD	Outer membrane usher protein FimD	Gly403Ala; Glu410Lys; Ser397Thr; Ile401Val	Unselected

Fiu	Catecholate siderophore receptor Fiu (Ferric iron uptake protein) (TonB-dependent receptor Fiu)	Thr493Ala	Unselected
gspD	Putative secretin GspD (Putative general secretion pathway protein D) (Putative type II secretion system protein D) (T2SS protein D)	Val627Ile	Gln617Leu
lolB	Outer-membrane lipoprotein LolB	Ala115Ser	Pro71Leu
lpoA	Penicillin-binding protein activator LpoA (PBP activator LpoA) (Lipoprotein activator of PBP from the outer membrane A)	Val106Ala; Ala96Thr; Ala292Val	Unselected
lpoB	Penicillin-binding protein activator LpoB (PBP activator LpoB) (Lipoprotein activator of PBP from the outer membrane B)	Ala115Ser	Unselected
Lpp	ATP-dependent Clp protease proteolytic subunit (EC 3.4.21.92) (Caseinolytic protease) (Endopeptidase Clp) (Heat shock protein F21.5) (Protease Ti)	Val26Ile	Unselected
mlaA	Intermembrane phospholipid transport system lipoprotein MlaA	Gly168Ser	Unselected
mliC	Membrane-bound lysozyme inhibitor of C-type lysozyme	His25Arg	Ala29Asp
mltB	Membrane-bound lytic murein transglycosylase B (EC 4.2.2.n1) (35 kDa soluble lytic transglycosylase) (Murein hydrolase B) (Slt35)	Lys324Arg; Asp64Glu; Val49Met	Unselected
nfrA	Bacteriophage adsorption protein A (Bacteriophage N4 adsorption protein A)	Ile784Leu	Ala115Asp; Ala98Asp
nlpD	Murein hydrolase activator NlpD	Ala228Thr	Unselected
nlpE	Lipoprotein NlpE (Copper homeostasis protein CutF)	His189Asn; Met172Ile; Ala29Thr	Unselected
ompC	Outer membrane porin C (Outer membrane protein 1B) (Outer membrane protein C) (Porin OmpC)	Thr305Val; Ile306Val	Unselected
ompF	Outer membrane porin F (Outer membrane protein 1A) (Outer membrane protein B) (Outer membrane protein F) (Outer membrane protein IA) (Porin OmpF)	Unselected	Val51Glu; Asp48Gly; Lys60Met
ompG	Outer membrane porin G (Outer membrane protein G)	Ala67Ser	Unselected
ompN	Outer membrane porin N (Outer membrane protein N) (Porin OmpN)	Unselected	Lys90Thr
pgaA	Poly-beta-1,6-N-acetyl-D-glucosamine export protein (PGA export protein) (Poly-beta-1,6-GlcNAc export protein)	Val26Ala; Ile18Leu; Gln11Lys; Thr150Ser; Ile106Val; Ile87Val	Ser90Arg; Pro451His; Pro129Ser; Phe599Val
pgaB	Poly-beta-1,6-N-acetyl-D-glucosamine N-deacetylase (PGA N-deacetylase) (Poly-beta-1,6-GlcNAc N-deacetylase) (EC 3.5.1.-)	Leu575Met	Unselected
slyB	Outer membrane lipoprotein SlyB	Val78Ile	Unselected

tam	Translocation and assembly module subunit TamA (Autotransporter assembly factor TamA)	Asn121Ser; Ala210Thr; Ile162Val	Leu216His; Gln123Leu;Ser158Pro
tsx	RNA polymerase sigma factor RpoS (Sigma S) (Sigma-38)	Ile53Leu	Unselected
uidC	Membrane-associated protein UidC	Unselected	Asn285Ile
yajI	Uncharacterized lipoprotein YajI	Glu164Asp	Asp167Gly
ycaL	Metalloprotease YcaL (EC 3.4.-.-)	Unselected	Ser158Arg
ccmA	Uncharacterized lipoprotein YceB	Unselected	Glu126Gly
yehB	Outer membrane usher protein YehB	Gln540His; Thr377Ser; Ile699Val	Unselected
yfaL	Probable autotransporter YfaL [Cleaved into: Probable secreted autotransporter protein YfaL; Probable autotransporter YfaL translocator]	Thr879Ala; Ser1091Asn; Thr919Asn; Glu1098Asp; Pro920Pro; Thr1101Val	Ser922Pro; Pro921Thr
yfeY	Uncharacterized protein YfeY	Gly142Ser	Arg167Ser

Table S2. The other part of genes encoding proteins connected with outer membrane (OM), flagellum, fimbria, lipopolysaccharide (LPS), exopolysaccharide (EPS) selected with mutfunc and list of conservative mutations selected with BLOSUM62.

Genes selected with mutfunc	Function	List of conservative mutations selected with BLOSUM62
ccmF	Cytochrome c-type biogenesis protein CcmF	Gly369Ala; Val387Ala; Val647Ala; Ala106Thr
cobS	Adenosylcobinamide-GDP ribazoletransferase (EC 2.7.8.26) (Cobalamin synthase) (Cobalamin-5'-phosphate synthase)	Unselected
creC	Sensor protein CreC (EC 2.7.13.3)	Ala363Ser; Ala401Thr; His399Tyr
fadI	3-ketoacyl-CoA thiolase FadI (EC 2.3.1.16) (ACSs) (Acetyl-CoA acyltransferase) (Acyl-CoA ligase) (Beta-ketothiolase) (Fatty acid oxidation complex subunit beta)	Val239Ala; Glu365Asp; Met431Ile; Phe238Leu; Val432Val; Leu433Val;
fdrA	Protein FdrA	Gly182Ala; Lys380Gln; Gly394Ser
fimF	Protein FimF	Glu142Asp; Ala62Thr
flgA	Flagella basal body P-ring formation protein FlgA	Gln29His; Ile39Val;
gsiA	Glutathione import ATP-binding protein GsiA (EC 7.4.2.10)	Thr122Ala
hyfB	Hydrogenase-4 component B (EC 1.-.-.-)	Ala262Val
kgtP	Alpha-ketoglutarate permease	Ala191Val; Ile179Val
msbA	Lipid A export ATP-binding/permease protein MsbA (EC 7.5.2.6)	Ser168Gly
phnC	Phosphonates import ATP-binding protein PhnC (EC 7.3.2.2)	Ile255Val

pstS	Phosphate-binding protein PstS (PBP)	Ile216Val
rseB	Sigma-E factor regulatory protein RseB	Thr273Ser
rsxD	Ion-translocating oxidoreductase complex subunit D (EC 7.-.-.-) (Rsx electron transport complex subunit D)	Leu313Met
sbmA	Peptide antibiotic transporter SbmA	Ile236Val
ttdT	L-tartrate/succinate antiporter (Tartrate carrier) (Tartrate transporter)	Val484Met; Ala457Val
yccM	Putative electron transport protein YccM	Val208Met
ydjE	Inner membrane metabolite transport protein YdjE	Thr331Ala; Val431Ile; Ile420MetLeu427Val
yejB	Inner membrane ABC transporter permease protein YejB	Asp96Asn
ygaP	Inner membrane protein YgaP	unselected
ynjC	Inner membrane ABC transporter permease protein YnjC	Val164Leu

Table S3. Regulatory genes selected with EcoCyc Database and list of conservative and non-conservative mutations selected with BLOSUM62. Mutations in bold were independently selected by mutfunc and are predicted to be impactful because of their presence in conservative regions[(*)] or possible destabilization of protein structure[(+)].

Regulatory genes obtained with EcoCyc Database	List of conservative mutations selected with BLOSUM62	List of non-conservative mutations selected with BLOSUM62	Connection with proteins detected in proteome analysis (2DE)
atoC	Gly283Ala	unselected	n/a
cbl	Glu254Lys	unselected	n/a
creB	unselected	Ala29Asp	n/a
deoR	Ile252Val	unselected	n/a
ecpR	Ile175Leu	unselected	Regulator gene of *fliC* (Table 6)
fliZ	Lys66Gln; Ala54Thr; Thr63Val	unselected	n/a
gadW	Thr2Ala	unselected	n/a
gadX	His130Gln; Ser88Thr; Ala75Val	Ser80Pro; Glu129Thr; Asn79Val	n/a
galR	unselected	Asp129Ala	Regulator gene of *mglB* (Table 6)
glnG	His331Arg	Gln289Leu	n/a
glrR	Asn269Asp; Ser74Gly; Ala285Thr	Glu100Ala; Leu28Arg	n/a

gntR	Asp198Glu; Ile156Val	unselected	n/a
hipA	Asn227Arg; **Glu234Lys**[*]; Ala242Thr; Ser424Thr; Tyr29Phe	unselected	n/a
kdpE	Tyr29Phe	unselected	n/a
lacI	Ala217Thr	Ala334Pro	n/a
lldR	Glu248Asp; **Asp124Glu**[†]	unselected	n/a
malI	Met139Ile	unselected	n/a
malT	Ile658Met	Pro706Ser	n/a
mlc	Thr167Ala; Arg311His	Gly138Asp; Ser162Pro	n/a
mlrA	Asn175Thr	unselected	n/a
modE	Glu193Asp; Ile162Val	unselected	Regulator gene of *oppA* (Table 7)
narL	Lys128Arg	unselected	n/a
nimR	unselected	Ser128Pro	n/a
phoP	Ile44Leu	unselected	n/a
pspF	**Leu106Phe**[*,†]	unselected	n/a
purR	Glu213Gln; Gln237Glu	Pro237Ser	n/a
rhaR	Val190Ile; Ala2Val	unselected	n/a
rne	unselected	Ile880Ala	n/a
rne	Val961Ala; His866Gln; Gln943His; Ile902Val	unselected	n/a
rpoD	Thr244Ala; Val229Ile	unselected	RNA polymerase sigma factor
rpoN	Glu150Asp; Ile165Met	unselected	n/a
rutR	Ile192Val	unselected	n/a
sdiA	His111Arg	unselected	n/a
sgrR	Asp219Asn	unselected	n/a
soxR	unselected	Gly74Arg	n/a
srlR	Ile198Val	unselected	n/a
tyrR	Ser53Thr	unselected	n/a
ydeO	Thr54Asn	unselected	n/a
ydeO	unselected	Asp20Tyr	n/a
yhaJ	unselected	Leu188Gln	n/a
