# Supplementary material for: How Bacteria Change after Exposure to Silver Nanoformulations: Analysis of the Genome and Outer Membrane Proteome
Source: Pathogens. 2021 Jun 29;10(7):817. doi: 10.3390/pathogens10070817 (PMC8308822; doi:10.3390/pathogens10070817)
Supplement: Supplementary file 1 [file pathogens-10-00817-s001.zip › supplementary file S1.zip!/Raporty (pod publikacje)/Reports (ATCC 11229)/Spot no. 1.pdf]

(MATRIX SCIENCE) Mascot Search Results

```
User            : sabina
Email           :
Search title    : E. coli_trypsin_100ppm
MS data file    : 281474976711861.mgf
Database        : SwissProt 2012_06 (536489 sequences; 190389898 residues)
Taxonomy        : Escherichia coli (22924 sequences)
Timestamp       : 21 Jan 2019 at 11:35:00 GMT
Protein hits    : OMPC_ECOLI Outer membrane protein C OS=Escherichia coli (strain K12) GN=ompC PE=1 SV=1
```

Mascot Score Histogram

Ions score is -10*Log(P), where P is the probability that the observed match is a random event.
Individual ions scores > 30 indicate identity or extensive homology (p<0.05).
Protein scores are derived from ions scores as a non-probabilistic basis for ranking protein hits.

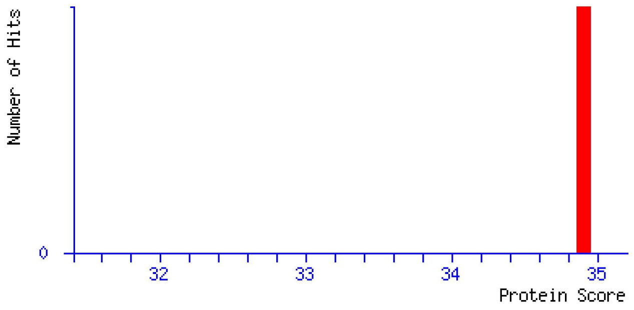

Peptide Summary Report

Format As [Peptide Summary] Help

Significance threshold p< [0.05] Max. number of hits [AUTO]

Standard scoring (●) MudPIT scoring ( ) Ions score or expect cut-off [15] Show sub-sets [0]

Show pop-ups (●) Suppress pop-ups ( ) Sort unassigned [Decreasing Score] Require bold red [ ]

[Select All] [Select None] [Search Selected] [ ] **Error tolerant** [Archive Report]

1. OMPC_ECOLI **Mass:** 40343 **Score:** 35 **Matches:** 2(0) **Sequences:** 2(0)
Outer membrane protein C OS=Escherichia coli (strain K12) GN=ompC PE=1 SV=1
[ ] Check to include this hit in error tolerant search or archive report

	Query	Observed	Mr(expt)	Mr(calc)	ppm	Miss	Score	Expect	Rank	Unique	Peptide
☑	4	1290.6073	1289.6000	1289.5677	25.1	0	18	1.3	1	U	K.FQDVGSFDYGR.N
☑	5	1348.7164	1347.7092	1347.6783	22.9	0	17	1.3	1	U	K.INLLDDNQFTR.D

Proteins matching the same set of peptides:
OMPC_ECO57 **Mass:** 40483 **Score:** 35 **Matches:** 2(0) **Sequences:** 2(0)
Outer membrane protein C OS=Escherichia coli O157:H7 GN=ompC PE=3 SV=1

Peptide matches not assigned to protein hits: (no details means no match)

	Query	Observed	Mr(expt)	Mr(calc)	ppm	Miss	Score	Expect	Rank	Unique	Peptide
☑	1	801.4739	800.4667	800.4392	34.3	0	13	3.9	1		GEVELVR
☑	3	868.4218	867.4145	866.4246	1142	0	9	6.5	1		SVTGNFSR
☑	2	*842.5388*	*841.5315*								
☑	6	*2973.8462*	*2972.8389*								

Search Parameters

```
Type of search         : MS/MS Ion Search
Enzyme                 : Trypsin
Fixed modifications    : Carboxymethyl (C)
Variable modifications : Oxidation (M)
Mass values            : Monoisotopic
Protein Mass           : Unrestricted
Peptide Mass Tolerance : ± 100 ppm (# 13C = 1)
Fragment Mass Tolerance: ± 1 Da
Max Missed Cleavages   : 2
Instrument type        : MALDI-TOF-TOF
Number of queries      : 6
```

Mascot: http://www.matrixscience.com/
