# Supplementary material for: How Bacteria Change after Exposure to Silver Nanoformulations: Analysis of the Genome and Outer Membrane Proteome
Source: Pathogens. 2021 Jun 29;10(7):817. doi: 10.3390/pathogens10070817 (PMC8308822; doi:10.3390/pathogens10070817)
Supplement: Supplementary file 1 [file pathogens-10-00817-s001.zip › supplementary file S1.zip!/Raporty (pod publikacje)/Reports (ATCC 11229)/Spot no. 2.pdf]

Mascot Search Results

```
User              : sabina
Email             :
Search title      : E. coli_trypsin_100ppm
MS data file      : 281474976711877.mgf
Database          : SwissProt 2012_06 (536489 sequences; 190389898 residues)
Taxonomy          : Escherichia coli (22924 sequences)
Timestamp         : 21 Jan 2019 at 12:27:01 GMT
Protein hits      : GLRX4_ECO57 Glutaredoxin-4 OS=Escherichia coli O157:H7 GN=grxD PE=3 SV=1
```

Mascot Score Histogram

Ions score is -10*Log(P), where P is the probability that the observed match is a random event.
Individual ions scores > 31 indicate identity or extensive homology (p<0.05).
Protein scores are derived from ions scores as a non-probabilistic basis for ranking protein hits.

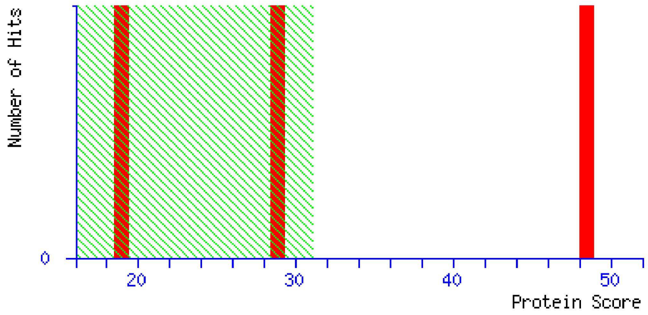

Peptide Summary Report

Format As | Peptide Summary | Help

Significance threshold p< 0.05 | Max. number of hits AUTO

Standard scoring (•) MudPIT scoring ( ) | Ions score or expect cut-off 15 | Show sub-sets 0

Show pop-ups (•) Suppress pop-ups ( ) | Sort unassigned Decreasing Score | Require bold red ☐

Select All | Select None | Search Selected | ☐ **Error tolerant** | Archive Report

1. GLRX4_ECO57 **Mass:** 13044 **Score:** 48 **Matches:** 1(1) **Sequences:** 1(1)
   Glutaredoxin-4 OS=Escherichia coli O157:H7 GN=grxD PE=3 SV=1
   ☐ Check to include this hit in error tolerant search or archive report

	Query	Observed	Mr(expt)	Mr(calc)	ppm	Miss	Score	Expect	Rank	Unique	Peptide
☑	1	1563.8765	1562.8692	1562.8093	38.3	0	48	0.001	1	U	R.FAYVDILQNPDIR.A

Proteins matching the same set of peptides:

GLRX4_ECOL6 **Mass:** 13044 **Score:** 48 **Matches:** 1(1) **Sequences:** 1(1)
Glutaredoxin-4 OS=Escherichia coli O6:H1 (strain CFT073 / ATCC 700928 / UPEC) GN=grxD PE=3 SV=1
GLRX4_ECOLI **Mass:** 13044 **Score:** 48 **Matches:** 1(1) **Sequences:** 1(1)
Glutaredoxin-4 OS=Escherichia coli (strain K12) GN=grxD PE=1 SV=1

Search Parameters

```
Type of search          : MS/MS Ion Search
Enzyme                  : Trypsin
Fixed modifications     : Carboxymethyl (C)
Variable modifications  : Oxidation (M)
Mass values             : Monoisotopic
Protein Mass            : Unrestricted
```

```
Peptide Mass Tolerance : ± 100 ppm (# 13C = 1)
Fragment Mass Tolerance: ± 1 Da
Max Missed Cleavages   : 2
Instrument type        : MALDI-TOF-TOF
Number of queries      : 1
```

Mascot: http://www.matrixscience.com/
