# Supplementary material for: How Bacteria Change after Exposure to Silver Nanoformulations: Analysis of the Genome and Outer Membrane Proteome
Source: Pathogens. 2021 Jun 29;10(7):817. doi: 10.3390/pathogens10070817 (PMC8308822; doi:10.3390/pathogens10070817)
Supplement: Supplementary file 1 [file pathogens-10-00817-s001.zip › supplementary file S1.zip!/Raporty (pod publikacje)/Reports (ATCC 11229)/Spot no. 3.pdf]

Mascot Search Results

User	:	sabina
Email	:	
Search title	:	E. coli_trypsin_100ppm
MS data file	:	281474976711866.mgf
Database	:	SwissProt 2012_06 (536489 sequences; 190389898 residues)
Taxonomy	:	Escherichia coli (22924 sequences)
Timestamp	:	21 Jan 2019 at 11:49:07 GMT
Protein hits	:	DGAL_ECOLI D-galactose-binding periplasmic protein OS=Escherichia coli (strain K12) GN=mglB PE=1 SV=1

Mascot Score Histogram

Ions score is -10*Log(P), where P is the probability that the observed match is a random event.
Individual ions scores > 30 indicate identity or extensive homology (p<0.05).
Protein scores are derived from ions scores as a non-probabilistic basis for ranking protein hits.

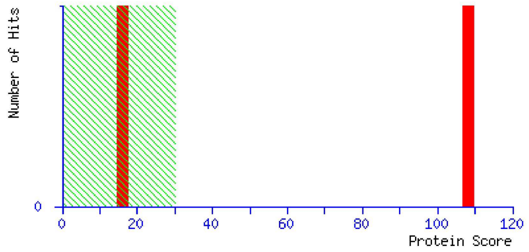

Peptide Summary Report

Format As | Peptide Summary | Help

Significance threshold p< 0.05 | Max. number of hits AUTO

Standard scoring ◉ MudPIT scoring ○ | Ions score or expect cut-off 15 | Show sub-sets 0

Show pop-ups ◉ Suppress pop-ups ○ | Sort unassigned Decreasing Score | Require bold red ☐

Select All | Select None | Search Selected | ☐ **Error tolerant** | Archive Report

1. DGAL_ECOLI **Mass:** 35690 **Score:** 108 **Matches:** 2(1) **Sequences:** 2(1)
D-galactose-binding periplasmic protein OS=Escherichia coli (strain K12) GN=mglB PE=1 SV=1
☐ Check to include this hit in error tolerant search or archive report

	Query	Observed	Mr(expt)	Mr(calc)	ppm	Miss	Score	Expect	Rank	Unique	Peptide
☑	2	1245.5616	1244.5544	1244.5496	3.85	0	24	0.2	1	U	K.YDDNFMSVVR.K
☑	4	1780.9161	1779.9089	1779.9043	2.56	1	84	2.5e-007	1	U	R.VPYVGVDKDNLAEFSK.K

Proteins matching the same set of peptides:
DGAL_ECOL6 **Mass:** 35690 **Score:** 108 **Matches:** 2(1) **Sequences:** 2(1)
D-galactose-binding periplasmic protein OS=Escherichia coli O6:H1 (strain CFT073 / ATCC 700928 / UPEC) GN=mglB PE=3 SV=1

Peptide matches not assigned to protein hits: (no details means no match)

	Query	Observed	Mr(expt)	Mr(calc)	ppm	Miss	Score	Expect	Rank	Unique	Peptide
☑	1	*842.5219*	*841.5146*								
☑	3	*1717.9191*	*1716.9118*								

Search Parameters

Type of search	:	MS/MS Ion Search
Enzyme	:	Trypsin
Fixed modifications	:	Carboxymethyl (C)
Variable modifications	:	Oxidation (M)
Mass values	:	Monoisotopic
Protein Mass	:	Unrestricted
Peptide Mass Tolerance	:	± 100 ppm (# ^{13}C = 1)
Fragment Mass Tolerance	:	± 1 Da
Max Missed Cleavages	:	2
Instrument type	:	MALDI-TOF-TOF
Number of queries	:	4
