# Supplementary material for: How Bacteria Change after Exposure to Silver Nanoformulations: Analysis of the Genome and Outer Membrane Proteome
Source: Pathogens. 2021 Jun 29;10(7):817. doi: 10.3390/pathogens10070817 (PMC8308822; doi:10.3390/pathogens10070817)
Supplement: Supplementary file 1 [file pathogens-10-00817-s001.zip › supplementary file S1.zip!/Raporty (pod publikacje)/Reports (ATCC 11229)/Spot no. 4.pdf]

Mascot Search Results

User : sabina
Email :
Search title : MS_32135_281474976711872
Database : SwissProt 2012_06 (536489 sequences; 190389898 residues)
Taxonomy : Escherichia coli (22924 sequences)
Timestamp : 21 Jan 2019 at 12:00:07 GMT
Top Score : 170 for MDH_ECO24, Malate dehydrogenase OS=Escherichia coli O139:H28 (strain E24377A / ETEC) GN=mdh PE=3 SV=1

Mascot Score Histogram

Protein score is -10*Log(P), where P is the probability that the observed match is a random event.
Protein scores greater than 56 are significant (p<0.05).

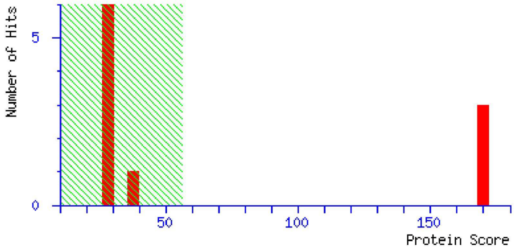

Concise Protein Summary Report

Format As | Concise Protein Summary | Help

Significance threshold p< 0.05 Max. number of hits AUTO

Re-Search All | Search Unmatched

1. MDH_ECO24 **Mass:** 32317 **Score:** 170 **Expect:** 2.3e-013 **Matches:** 16
Malate dehydrogenase OS=Escherichia coli O139:H28 (strain E24377A / ETEC) GN=mdh PE=3 SV=1
MDH_ECO27 **Mass:** 32317 **Score:** 170 **Expect:** 2.3e-013 **Matches:** 16
Malate dehydrogenase OS=Escherichia coli O127:H6 (strain E2348/69 / EPEC) GN=mdh PE=3 SV=1
MDH_ECO45 **Mass:** 32317 **Score:** 170 **Expect:** 2.3e-013 **Matches:** 16
Malate dehydrogenase OS=Escherichia coli O45:K1 (strain S88 / ExPEC) GN=mdh PE=3 SV=1
MDH_ECO57 **Mass:** 32317 **Score:** 170 **Expect:** 2.3e-013 **Matches:** 16
Malate dehydrogenase OS=Escherichia coli O157:H7 GN=mdh PE=3 SV=1
MDH_ECO5E **Mass:** 32317 **Score:** 170 **Expect:** 2.3e-013 **Matches:** 16
Malate dehydrogenase OS=Escherichia coli O157:H7 (strain EC4115 / EHEC) GN=mdh PE=3 SV=1
MDH_ECO81 **Mass:** 32317 **Score:** 170 **Expect:** 2.3e-013 **Matches:** 16
Malate dehydrogenase OS=Escherichia coli O81 (strain ED1a) GN=mdh PE=3 SV=1
MDH_ECOBW **Mass:** 32317 **Score:** 170 **Expect:** 2.3e-013 **Matches:** 16
Malate dehydrogenase OS=Escherichia coli (strain K12 / MC4100 / BW2952) GN=mdh PE=3 SV=1
MDH_ECODH **Mass:** 32317 **Score:** 170 **Expect:** 2.3e-013 **Matches:** 16
Malate dehydrogenase OS=Escherichia coli (strain K12 / DH10B) GN=mdh PE=3 SV=1
MDH_ECOHS **Mass:** 32317 **Score:** 170 **Expect:** 2.3e-013 **Matches:** 16
Malate dehydrogenase OS=Escherichia coli O9:H4 (strain HS) GN=mdh PE=3 SV=1
MDH_ECOK1 **Mass:** 32317 **Score:** 170 **Expect:** 2.3e-013 **Matches:** 16
Malate dehydrogenase OS=Escherichia coli O1:K1 / APEC GN=mdh PE=3 SV=1
MDH_ECOL6 **Mass:** 32317 **Score:** 170 **Expect:** 2.3e-013 **Matches:** 16
Malate dehydrogenase OS=Escherichia coli O6:H1 (strain CFT073 / ATCC 700928 / UPEC) GN=mdh PE=3 SV=1
MDH_ECOLI **Mass:** 32317 **Score:** 170 **Expect:** 2.3e-013 **Matches:** 16
Malate dehydrogenase OS=Escherichia coli (strain K12) GN=mdh PE=1 SV=1
MDH_ECOUT **Mass:** 32317 **Score:** 170 **Expect:** 2.3e-013 **Matches:** 16
Malate dehydrogenase OS=Escherichia coli (strain UTI89 / UPEC) GN=mdh PE=3 SV=2
MDH_ECOSE **Mass:** 32333 **Score:** 155 **Expect:** 7.2e-012 **Matches:** 15
Malate dehydrogenase OS=Escherichia coli (strain SE11) GN=mdh PE=3 SV=1
MDH_ECO7I **Mass:** 32317 **Score:** 154 **Expect:** 9.1e-012 **Matches:** 15
Malate dehydrogenase OS=Escherichia coli O7:K1 (strain IAI39 / ExPEC) GN=mdh PE=3 SV=1
MDH_ECOSM **Mass:** 32317 **Score:** 154 **Expect:** 9.1e-012 **Matches:** 15
Malate dehydrogenase OS=Escherichia coli (strain SMS-3-5 / SECEC) GN=mdh PE=3 SV=1
MDH_ECOLC **Mass:** 32307 **Score:** 154 **Expect:** 9.1e-012 **Matches:** 15
Malate dehydrogenase OS=Escherichia coli (strain ATCC 8739 / DSM 1576 / Crooks) GN=mdh PE=3 SV=1
MDH_ECOLU **Mass:** 32316 **Score:** 139 **Expect:** 2.9e-010 **Matches:** 14
Malate dehydrogenase OS=Escherichia coli O17:K52:H18 (strain UMN026 / ExPEC) GN=mdh PE=3 SV=1

---

2. MDH_ECO55 **Mass:** 32290 **Score:** 170 **Expect:** 2.3e-013 **Matches:** 16
Malate dehydrogenase OS=Escherichia coli (strain 55989 / EAEC) GN=mdh PE=3 SV=1
MDH_ECO8A **Mass:** 32290 **Score:** 170 **Expect:** 2.3e-013 **Matches:** 16
Malate dehydrogenase OS=Escherichia coli O8 (strain IAI1) GN=mdh PE=3 SV=1

---

3. MDH_ECOL5 **Mass:** 32331 **Score:** 170 **Expect:** 2.3e-013 **Matches:** 16

Malate dehydrogenase OS=Escherichia coli O6:K15:H31 (strain 536 / UPEC) GN=mdh PE=3 SV=1

---

4. LFTR_ECO27 **Mass:** 26714 **Score:** 36 **Expect:** 6.5 **Matches:** 5
Leucyl/phenylalanyl-tRNA--protein transferase OS=Escherichia coli O127:H6 (strain E2348/69 / EPEC) GN=aat PE=3 SV=1

Search Parameters

Type of search : Peptide Mass Fingerprint
Enzyme : Trypsin
Variable modifications : Carbamidomethyl (C),Oxidation (M)
Mass values : Monoisotopic
Protein Mass : Unrestricted
Peptide Mass Tolerance : ± 100 ppm
Peptide Charge State : 1+
Max Missed Cleavages : 1
Number of queries : 45

Mascot: http://www.matrixscience.com/
