# Supplementary material for: How Bacteria Change after Exposure to Silver Nanoformulations: Analysis of the Genome and Outer Membrane Proteome
Source: Pathogens. 2021 Jun 29;10(7):817. doi: 10.3390/pathogens10070817 (PMC8308822; doi:10.3390/pathogens10070817)
Supplement: Supplementary file 1 [file pathogens-10-00817-s001.zip › supplementary file S1.zip!/Raporty (pod publikacje)/Reports (ATCC 11229)/Spot no. 5.pdf]

MATRIX SCIENCE Mascot Search Results

**User**	:	**sabina**
**Email**	:	
**Search title**	:	**E. coli_trypsin_100ppm**
**MS data file**	:	**281474976711881.mgf**
**Database**	:	**SwissProt 2012_06 (536489 sequences; 190389898 residues)**
**Taxonomy**	:	**Escherichia coli (22924 sequences)**
**Timestamp**	:	**21 Jan 2019 at 13:43:26 GMT**
**Protein hits**	:	**OPPA_ECOLI** Periplasmic oligopeptide-binding protein OS=Escherichia coli (strain K12) GN=oppA PE=1 SV=2

Mascot Score Histogram

Ions score is -10*Log(P), where P is the probability that the observed match is a random event.
Individual ions scores > 30 indicate identity or extensive homology (p<0.05).
Protein scores are derived from ions scores as a non-probabilistic basis for ranking protein hits.

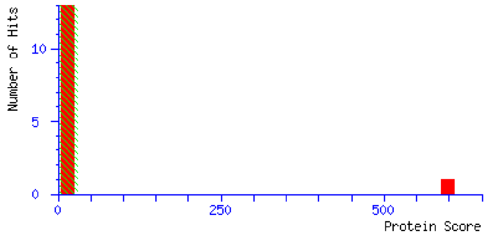

Peptide Summary Report

Format As [Peptide Summary] Help

Significance threshold p< [0.05] Max. number of hits [AUTO]

Standard scoring ◉ MudPIT scoring ○ Ions score or expect cut-off [15] Show sub-sets [0]

Show pop-ups ◉ Suppress pop-ups ○ Sort unassigned [Decreasing Score] Require bold red ☐

Select All | Select None | Search Selected | ☐ **Error tolerant** | Archive Report

1. OPPA_ECOLI **Mass:** 60977 **Score:** 598 **Matches:** 5(5) **Sequences:** 5(5)
Periplasmic oligopeptide-binding protein OS=Escherichia coli (strain K12) GN=oppA PE=1 SV=2
☐ Check to include this hit in error tolerant search or archive report

	Query	Observed	Mr(expt)	Mr(calc)	ppm	Miss	Score	Expect	Rank	Unique	Peptide
☑	1	958.5591	957.5519	957.5185	34.9	0	41	0.0071	1	U	K.VWTFHLR.K
☑	2	2143.0947	2142.0875	2141.9807	49.8	0	133	3.4e-012	1	U	K.WSDGTPVTAQDFVYSWQR.S
☑	3	2232.2847	2231.2774	2231.1798	43.8	0	155	2e-014	1	U	K.TVINQVTYLPIASEVTDVNR.Y
☑	4	2442.3635	2441.3562	2441.2227	54.7	1	146	1.5e-013	1	U	K.AEQQLDKDSAIVPVYYYVNAR.L
☑	5	2662.4866	2661.4793	2660.3347	430	1	126	1.5e-011	1	U	K.WTQPGNIVTNGAYTLKDWVVNER.I

Peptide matches not assigned to protein hits: (no details means no match)

	Query	Observed	Mr(expt)	Mr(calc)	ppm	Miss	Score	Expect	Rank	Unique	Peptide
☑	6	3727.8831	3726.8758	3725.7035	315	1	11	3.6	1		SDNGASNLLRLPPASSSTTSGASNTSSTSQGDIMDQR + Oxidation (M)

Search Parameters

**Type of search**	:	**MS/MS Ion Search**
**Enzyme**	:	**Trypsin**
**Fixed modifications**	:	**Carboxymethyl (C)**
**Variable modifications**	:	**Oxidation (M)**
**Mass values**	:	**Monoisotopic**
**Protein Mass**	:	**Unrestricted**
**Peptide Mass Tolerance**	:	**± 100 ppm (# ^{13}C = 1)**
**Fragment Mass Tolerance**	:	**± 1 Da**
**Max Missed Cleavages**	:	**2**
**Instrument type**	:	**MALDI-TOF-TOF**
**Number of queries**	:	**6**
