# Supplementary material for: How Bacteria Change after Exposure to Silver Nanoformulations: Analysis of the Genome and Outer Membrane Proteome
Source: Pathogens. 2021 Jun 29;10(7):817. doi: 10.3390/pathogens10070817 (PMC8308822; doi:10.3390/pathogens10070817)
Supplement: Supplementary file 1 [file pathogens-10-00817-s001.zip › supplementary file S1.zip!/Raporty (pod publikacje)/Reports (ATCC 11229)/Spot no. 6.pdf]

{MATRIX SCIENCE} Mascot Search Results

```
User            : sabina
Email           :
Search title    : E. coli_trypsin_100ppm
MS data file    : 281474976711885.mgf
Database        : SwissProt 2012_06 (536489 sequences; 190389898 residues)
Taxonomy        : Escherichia coli (22924 sequences)
Timestamp       : 21 Jan 2019 at 15:03:50 GMT
Protein hits    : OPPA_ECOLI Periplasmic oligopeptide-binding protein OS=Escherichia coli (strain K12) GN=oppA PE=1 SV=2
```

Mascot Score Histogram

Ions score is -10*Log(P), where P is the probability that the observed match is a random event.
Individual ions scores > 31 indicate identity or extensive homology (p<0.05).
Protein scores are derived from ions scores as a non-probabilistic basis for ranking protein hits.

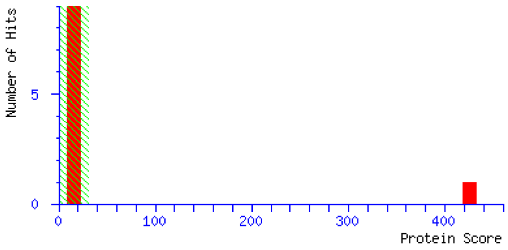

Peptide Summary Report

Format As [Peptide Summary] Help

Significance threshold p< [0.05] Max. number of hits [AUTO]

Standard scoring ◉ MudPIT scoring ○ Ions score or expect cut-off [15] Show sub-sets [0]

Show pop-ups ◉ Suppress pop-ups ○ Sort unassigned [Decreasing Score] Require bold red ☐

[Select All] [Select None] [Search Selected] ☐ **Error tolerant** [Archive Report]

1. OPPA_ECOLI **Mass:** 60977 **Score:** 426 **Matches:** 4(3) **Sequences:** 4(3)
Periplasmic oligopeptide-binding protein OS=Escherichia coli (strain K12) GN=oppA PE=1 SV=2
☐ Check to include this hit in error tolerant search or archive report

	Query	Observed	Mr(expt)	Mr(calc)	ppm	Miss	Score	Expect	Rank	Unique	Peptide
☑	1	958.5201	957.5129	957.5185	-5.86	0	21	0.72	1	U	K.VWTFHLR.K
☑	2	2143.0610	2142.0538	2141.9807	34.1	0	131	4.8e-012	1	U	K.WSDGTPVTAQDFVYSWQR.S
☑	3	2232.2498	2231.2425	2231.1798	28.1	0	164	2.8e-015	1	U	K.TVINQVTYLPIASEVTDVNR.Y
☑	4	2442.3220	2441.3147	2441.2227	37.7	1	110	6.5e-010	1	U	K.AEQQLDKDSAIVPVYYYVNAR.L

Peptide matches not assigned to protein hits: (no details means no match)

	Query	Observed	Mr(expt)	Mr(calc)	ppm	Miss	Score	Expect	Rank	Unique	Peptide
☑	5	3727.8269	3726.8196	3726.7586	16.4	1	12	2.5	1		LIEQIDEYLDDTFMLFSSYGINMQDLQKWR + Oxidation (M)

Search Parameters

```
Type of search          : MS/MS Ion Search
Enzyme                  : Trypsin
Fixed modifications     : Carboxymethyl (C)
Variable modifications  : Oxidation (M)
Mass values             : Monoisotopic
Protein Mass            : Unrestricted
Peptide Mass Tolerance  : ± 100 ppm (# 13C = 1)
Fragment Mass Tolerance : ± 1 Da
Max Missed Cleavages    : 2
Instrument type         : MALDI-TOF-TOF
Number of queries       : 5
```
