# Supplementary material for: How Bacteria Change after Exposure to Silver Nanoformulations: Analysis of the Genome and Outer Membrane Proteome
Source: Pathogens. 2021 Jun 29;10(7):817. doi: 10.3390/pathogens10070817 (PMC8308822; doi:10.3390/pathogens10070817)
Supplement: Supplementary file 1 [file pathogens-10-00817-s001.zip › supplementary file S1.zip!/Raporty (pod publikacje)/Reports (ATCC 11229)/Spot no. 7.pdf]

Mascot Search Results

User : sabina
Email :
Search title : E. coli_trypsin_100ppm
MS data file : 281474976711871.mgf
Database : SwissProt 2012_06 (536489 sequences; 190389898 residues)
Taxonomy : Escherichia coli (22924 sequences)
Timestamp : 21 Jan 2019 at 11:57:23 GMT
Protein hits : CYSP_ECOLI Thiosulfate-binding protein OS=Escherichia coli (strain K12) GN=cysP PE=1 SV=1

Mascot Score Histogram

Ions score is -10*Log(P), where P is the probability that the observed match is a random event.
Individual ions scores > 30 indicate identity or extensive homology ($p<0.05$).
Protein scores are derived from ions scores as a non-probabilistic basis for ranking protein hits.

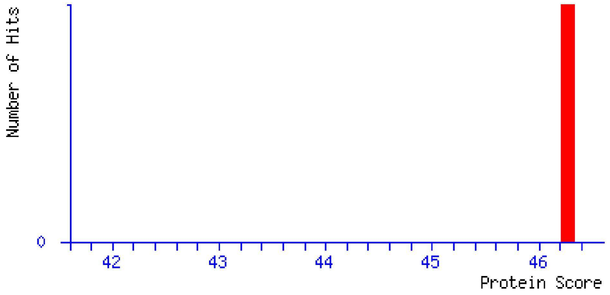

Peptide Summary Report

Format As | Peptide Summary | Help

Significance threshold p< 0.05 | Max. number of hits AUTO

Standard scoring (selected) | MudPIT scoring | Ions score or expect cut-off 15 | Show sub-sets 0

Show pop-ups (selected) | Suppress pop-ups | Sort unassigned Decreasing Score | Require bold red

Select All | Select None | Search Selected | **Error tolerant** | Archive Report

1. CYSP_ECOLI **Mass:** 37591 **Score:** 46 **Matches:** 1(1) **Sequences:** 1(1)
Thiosulfate-binding protein OS=Escherichia coli (strain K12) GN=cysP PE=1 SV=1
Check to include this hit in error tolerant search or archive report

	Query	Observed	Mr(expt)	Mr(calc)	ppm	Miss	Score	Expect	Rank	Unique	Peptide
☑	2	1888.9502	1887.9429	1887.9519	-4.76	0	46	0.0015	1	U	R.ELFAALNPPFEQQWAK.D

Peptide matches not assigned to protein hits: (no details means no match)

	Query	Observed	Mr(expt)	Mr(calc)	ppm	Miss	Score	Expect	Rank	Unique	Peptide
☑	1	842.5319	841.5246	841.4406	99.8	1	13	2.4	1		RDDAPLR
☑	3	2640.5410	2639.5337	2638.2843	474	1	10	4.5	1		TCHAAIIARELGIPAVVGCGDATER

Search Parameters

Type of search : MS/MS Ion Search
Enzyme : Trypsin
Fixed modifications : Carboxymethyl (C)
Variable modifications : Oxidation (M)
Mass values : Monoisotopic
Protein Mass : Unrestricted
Peptide Mass Tolerance : ± 100 ppm (# ^{13}C = 1)
Fragment Mass Tolerance: ± 1 Da
Max Missed Cleavages : 2
Instrument type : MALDI-TOF-TOF
Number of queries : 3

Mascot: http://www.matrixscience.com/
