# Supplementary material for: How Bacteria Change after Exposure to Silver Nanoformulations: Analysis of the Genome and Outer Membrane Proteome
Source: Pathogens. 2021 Jun 29;10(7):817. doi: 10.3390/pathogens10070817 (PMC8308822; doi:10.3390/pathogens10070817)
Supplement: Supplementary file 1 [file pathogens-10-00817-s001.zip › supplementary file S1.zip!/Raporty (pod publikacje)/Reports (BW25113)/Spot no. 10.pdf]

# Mascot Search Results

```
User          : sabina
Email         :
Search title  : MS_33223_281474976711921
Database      : SwissProt 2012_06 (536489 sequences; 190389898 residues)
Taxonomy      : Escherichia coli (22924 sequences)
Timestamp     : 18 Feb 2019 at 13:12:43 GMT
Top Score     : 62 for CUTC_ECOHS, Copper homeostasis protein CutC OS=Escherichia coli O9:H4 (strain HS) GN=cutC PE=3 SV=1
```

## Mascot Score Histogram

Protein score is -10*Log(P), where P is the probability that the observed match is a random event.
Protein scores greater than 56 are significant (p<0.05).

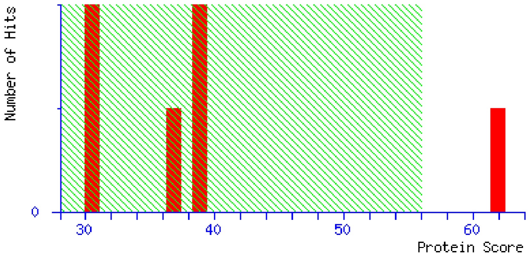


## Concise Protein Summary Report

Format As | Concise Protein Summary | Help

Significance threshold p< 0.05   Max. number of hits AUTO

Re-Search All | Search Unmatched

1. CUTC_ECOHS **Mass:** 26623 **Score:** 62 **Expect:** 0.015 **Matches:** 7
Copper homeostasis protein CutC OS=Escherichia coli O9:H4 (strain HS) GN=cutC PE=3 SV=1
CUTC_ECOLC **Mass:** 26623 **Score:** 62 **Expect:** 0.015 **Matches:** 7
Copper homeostasis protein CutC OS=Escherichia coli (strain ATCC 8739 / DSM 1576 / Crooks) GN=cutC PE=3 SV=1
CUTC_ECO24 **Mass:** 26744 **Score:** 50 **Expect:** 0.24 **Matches:** 6
Copper homeostasis protein CutC OS=Escherichia coli O139:H28 (strain E24377A / ETEC) GN=cutC PE=3 SV=1
CUTC_ECO27 **Mass:** 26653 **Score:** 50 **Expect:** 0.24 **Matches:** 6
Copper homeostasis protein CutC OS=Escherichia coli O127:H6 (strain E2348/69 / EPEC) GN=cutC PE=3 SV=1
CUTC_ECO55 **Mass:** 26686 **Score:** 50 **Expect:** 0.24 **Matches:** 6
Copper homeostasis protein CutC OS=Escherichia coli (strain 55989 / EAEC) GN=cutC PE=3 SV=1
CUTC_ECO8A **Mass:** 26744 **Score:** 50 **Expect:** 0.24 **Matches:** 6
Copper homeostasis protein CutC OS=Escherichia coli O8 (strain IAI1) GN=cutC PE=3 SV=1
CUTC_ECOBW **Mass:** 26744 **Score:** 50 **Expect:** 0.24 **Matches:** 6
Copper homeostasis protein CutC OS=Escherichia coli (strain K12 / MC4100 / BW2952) GN=cutC PE=3 SV=1
CUTC_ECODH **Mass:** 26744 **Score:** 50 **Expect:** 0.24 **Matches:** 6
Copper homeostasis protein CutC OS=Escherichia coli (strain K12 / DH10B) GN=cutC PE=3 SV=1
CUTC_ECOL5 **Mass:** 26653 **Score:** 50 **Expect:** 0.24 **Matches:** 6
Copper homeostasis protein CutC OS=Escherichia coli O6:K15:H31 (strain 536 / UPEC) GN=cutC PE=3 SV=1
CUTC_ECOL6 **Mass:** 26653 **Score:** 50 **Expect:** 0.24 **Matches:** 6
Copper homeostasis protein CutC OS=Escherichia coli O6:H1 (strain CFT073 / ATCC 700928 / UPEC) GN=cutC PE=3 SV=1
CUTC_ECOLI **Mass:** 26744 **Score:** 50 **Expect:** 0.24 **Matches:** 6
Copper homeostasis protein CutC OS=Escherichia coli (strain K12) GN=cutC PE=3 SV=1
CUTC_ECOSE **Mass:** 26744 **Score:** 50 **Expect:** 0.24 **Matches:** 6
Copper homeostasis protein CutC OS=Escherichia coli (strain SE11) GN=cutC PE=3 SV=1
CUTC_ECO7I **Mass:** 26510 **Score:** 38 **Expect:** 3.4 **Matches:** 5
Copper homeostasis protein CutC OS=Escherichia coli O7:K1 (strain IAI39 / ExPEC) GN=cutC PE=3 SV=1
CUTC_ECOLU **Mass:** 26544 **Score:** 38 **Expect:** 3.4 **Matches:** 5
Copper homeostasis protein CutC OS=Escherichia coli O17:K52:H18 (strain UMN026 / ExPEC) GN=cutC PE=3 SV=1
CUTC_ECOSM **Mass:** 26568 **Score:** 38 **Expect:** 3.4 **Matches:** 5
Copper homeostasis protein CutC OS=Escherichia coli (strain SMS-3-5 / SECEC) GN=cutC PE=3 SV=1

---

2. LYS4_ECOLX **Mass:** 5629 **Score:** 39 **Expect:** 3 **Matches:** 4
Lysis protein for colicin N OS=Escherichia coli GN=cnl PE=1 SV=1

---

## Search Parameters

```
Type of search           : Peptide Mass Fingerprint
Enzyme                   : Trypsin
Variable modifications   : Carbamidomethyl (C),Oxidation (M)
Mass values              : Monoisotopic
Protein Mass             : Unrestricted
```

```
Peptide Mass Tolerance : ± 100 ppm
Peptide Charge State   : 1+
Max Missed Cleavages   : 1
Number of queries      : 33
```

| **Mascot:** http://www.matrixscience.com/ |
|---|
