# Supplementary material for: How Bacteria Change after Exposure to Silver Nanoformulations: Analysis of the Genome and Outer Membrane Proteome
Source: Pathogens. 2021 Jun 29;10(7):817. doi: 10.3390/pathogens10070817 (PMC8308822; doi:10.3390/pathogens10070817)
Supplement: Supplementary file 1 [file pathogens-10-00817-s001.zip › supplementary file S1.zip!/Raporty (pod publikacje)/Reports (BW25113)/Spot no. 2.pdf]

Mascot Search Results

User : sabina
Email :
Search title : MS_33319_281474976711925
Database : SwissProt 2012_06 (536489 sequences; 190389898 residues)
Taxonomy : Escherichia coli (22924 sequences)
Timestamp : 18 Feb 2019 at 13:19:00 GMT
Top Score : 82 for FLIC_ECOLI, Flagellin OS=Escherichia coli (strain K12) GN=fliC PE=1 SV=2

Mascot Score Histogram

Protein score is -10*Log(P), where P is the probability that the observed match is a random event.
Protein scores greater than 56 are significant (p<0.05).

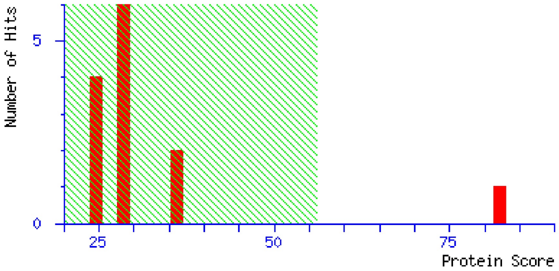

Concise Protein Summary Report

Format As | Concise Protein Summary | Help

Significance threshold p< 0.05 Max. number of hits AUTO

Re-Search All | Search Unmatched

1. FLIC_ECOLI **Mass:** 51265 **Score:** 82 **Expect:** 0.00014 **Matches:** 10
   Flagellin OS=Escherichia coli (strain K12) GN=fliC PE=1 SV=2

---

2. TRUC_ECO57 **Mass:** 29690 **Score:** 36 **Expect:** 5.5 **Matches:** 5
   tRNA pseudouridine synthase C OS=Escherichia coli O157:H7 GN=truC PE=3 SV=1
   TRUC_ECOL6 **Mass:** 29589 **Score:** 36 **Expect:** 5.5 **Matches:** 5
   tRNA pseudouridine synthase C OS=Escherichia coli O6:H1 (strain CFT073 / ATCC 700928 / UPEC) GN=truC PE=3 SV=1
   TRUC_ECOLI **Mass:** 29647 **Score:** 36 **Expect:** 5.5 **Matches:** 5
   tRNA pseudouridine synthase C OS=Escherichia coli (strain K12) GN=truC PE=1 SV=1

Search Parameters

Type of search : Peptide Mass Fingerprint
Enzyme : Trypsin
Variable modifications : Carbamidomethyl (C),Oxidation (M)
Mass values : Monoisotopic
Protein Mass : Unrestricted
Peptide Mass Tolerance : ± 100 ppm
Peptide Charge State : 1+
Max Missed Cleavages : 1
Number of queries : 36
Selected for scoring : 24
