# Supplementary material for: How Bacteria Change after Exposure to Silver Nanoformulations: Analysis of the Genome and Outer Membrane Proteome
Source: Pathogens. 2021 Jun 29;10(7):817. doi: 10.3390/pathogens10070817 (PMC8308822; doi:10.3390/pathogens10070817)
Supplement: Supplementary file 1 [file pathogens-10-00817-s001.zip › supplementary file S1.zip!/Raporty (pod publikacje)/Reports (BW25113)/Spot no. 5.pdf]

Mascot Search Results

User : sabina
Email :
Search title : MS_33199_281474976711920
Database : SwissProt 2012_06 (536489 sequences; 190389898 residues)
Taxonomy : Escherichia coli (22924 sequences)
Timestamp : 18 Feb 2019 at 13:06:43 GMT
Top Score : 127 for DNAK_ECO24, Chaperone protein DnaK OS=Escherichia coli O139:H28 (strain E24377A / ETEC) GN=dnaK PE=3 SV=1

Mascot Score Histogram

Protein score is -10*Log(P), where P is the probability that the observed match is a random event.
Protein scores greater than 56 are significant (p<0.05).

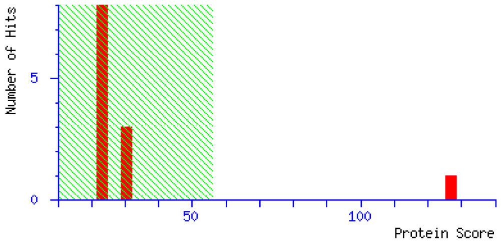

Concise Protein Summary Report

Format As | Concise Protein Summary | Help

Significance threshold p< 0.05 Max. number of hits AUTO

Re-Search All | Search Unmatched

1. DNAK_ECO24 **Mass:** 69072 **Score:** 127 **Expect:** 4.6e-009 **Matches:** 15
Chaperone protein DnaK OS=Escherichia coli O139:H28 (strain E24377A / ETEC) GN=dnaK PE=3 SV=1
DNAK_ECO57 **Mass:** 69072 **Score:** 127 **Expect:** 4.6e-009 **Matches:** 15
Chaperone protein DnaK OS=Escherichia coli O157:H7 GN=dnaK PE=3 SV=2
DNAK_ECOHS **Mass:** 69088 **Score:** 127 **Expect:** 4.6e-009 **Matches:** 15
Chaperone protein DnaK OS=Escherichia coli O9:H4 (strain HS) GN=dnaK PE=3 SV=1
DNAK_ECOK1 **Mass:** 69072 **Score:** 127 **Expect:** 4.6e-009 **Matches:** 15
Chaperone protein DnaK OS=Escherichia coli O1:K1 / APEC GN=dnaK PE=3 SV=1
DNAK_ECOL5 **Mass:** 69072 **Score:** 127 **Expect:** 4.6e-009 **Matches:** 15
Chaperone protein DnaK OS=Escherichia coli O6:K15:H31 (strain 536 / UPEC) GN=dnaK PE=3 SV=1
DNAK_ECOL6 **Mass:** 69072 **Score:** 127 **Expect:** 4.6e-009 **Matches:** 15
Chaperone protein DnaK OS=Escherichia coli O6:H1 (strain CFT073 / ATCC 700928 / UPEC) GN=dnaK PE=3 SV=2
DNAK_ECOLC **Mass:** 69072 **Score:** 127 **Expect:** 4.6e-009 **Matches:** 15
Chaperone protein DnaK OS=Escherichia coli (strain ATCC 8739 / DSM 1576 / Crooks) GN=dnaK PE=3 SV=1
DNAK_ECOLI **Mass:** 69072 **Score:** 127 **Expect:** 4.6e-009 **Matches:** 15
Chaperone protein DnaK OS=Escherichia coli (strain K12) GN=dnaK PE=1 SV=2
DNAK_ECOUT **Mass:** 69072 **Score:** 127 **Expect:** 4.6e-009 **Matches:** 15
Chaperone protein DnaK OS=Escherichia coli (strain UTI89 / UPEC) GN=dnaK PE=3 SV=1
MTE5_ECOLX **Mass:** 34617 **Score:** 32 **Expect:** 15 **Matches:** 5
Modification methylase EcoRV OS=Escherichia coli GN=ecoRVM PE=3 SV=1
ACPS_ECOLU **Mass:** 14102 **Score:** 27 **Expect:** 46 **Matches:** 3
Holo-[acyl-carrier-protein] synthase OS=Escherichia coli O17:K52:H18 (strain UMN026 / ExPEC) GN=acpS PE=3 SV=1
ACPS_ECOSM **Mass:** 14102 **Score:** 27 **Expect:** 46 **Matches:** 3
Holo-[acyl-carrier-protein] synthase OS=Escherichia coli (strain SMS-3-5 / SECEC) GN=acpS PE=3 SV=1
YGAU_ECOL6 **Mass:** 16053 **Score:** 24 **Expect:** 87 **Matches:** 3
Uncharacterized protein ygaU OS=Escherichia coli O6:H1 (strain CFT073 / ATCC 700928 / UPEC) GN=ygaU PE=3 SV=2
YGAU_ECOLI **Mass:** 16053 **Score:** 24 **Expect:** 87 **Matches:** 3
Uncharacterized protein ygaU OS=Escherichia coli (strain K12) GN=ygaU PE=1 SV=2

---

2. YCGJ_ECOLI **Mass:** 13405 **Score:** 32 **Expect:** 13 **Matches:** 5
Uncharacterized protein ycgJ OS=Escherichia coli (strain K12) GN=ycgJ PE=4 SV=1

Search Parameters

Type of search : Peptide Mass Fingerprint
Enzyme : Trypsin
Variable modifications : Carbamidomethyl (C),Oxidation (M)
Mass values : Monoisotopic
Protein Mass : Unrestricted
Peptide Mass Tolerance : ± 100 ppm
Peptide Charge State : 1+
Max Missed Cleavages : 1
Number of queries : 34
