# Supplementary material for: How Bacteria Change after Exposure to Silver Nanoformulations: Analysis of the Genome and Outer Membrane Proteome
Source: Pathogens. 2021 Jun 29;10(7):817. doi: 10.3390/pathogens10070817 (PMC8308822; doi:10.3390/pathogens10070817)
Supplement: Supplementary file 1 [file pathogens-10-00817-s001.zip › supplementary file S1.zip!/Raporty (pod publikacje)/Reports (BW25113)/Spot no. 6.pdf]

# Mascot Search Results

| | | |
|---|---|---|
| **User** | : | **sabina** |
| **Email** | : | |
| **Search title** | : | **E. coli_trypsin_100ppm** |
| **MS data file** | : | **281474976711944.mgf** |
| **Database** | : | **SwissProt 2012_06 (536489 sequences; 190389898 residues)** |
| **Taxonomy** | : | **Escherichia coli (22924 sequences)** |
| **Timestamp** | : | **19 Feb 2019 at 10:38:19 GMT** |
| **Protein hits** | : | ACEA_ECOLI Isocitrate lyase OS=Escherichia coli (strain K12) GN=aceA PE=1 SV=1 |

## Mascot Score Histogram

Ions score is -10*Log(P), where P is the probability that the observed match is a random event.
Individual ions scores > 30 indicate identity or extensive homology (p<0.05).
Protein scores are derived from ions scores as a non-probabilistic basis for ranking protein hits.

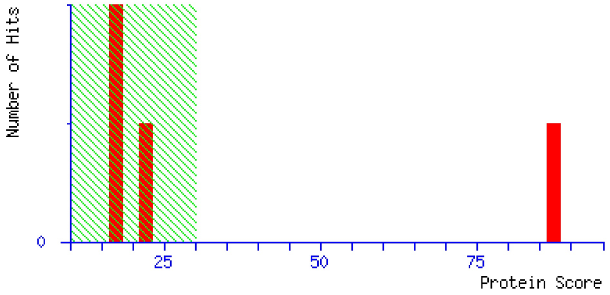


## Peptide Summary Report

Format As | Peptide Summary | Help

Significance threshold p< 0.05 | Max. number of hits AUTO

Standard scoring ◉ MudPIT scoring ○ | Ions score or expect cut-off 15 | Show sub-sets 0

Show pop-ups ◉ Suppress pop-ups ○ | Sort unassigned Decreasing Score | Require bold red ☐

Select All | Select None | Search Selected | ☐ **Error tolerant** | Archive Report

1. ACEA_ECOLI **Mass:** 47782 **Score:** 87 **Matches:** 2(1) **Sequences:** 2(1)
Isocitrate lyase OS=Escherichia coli (strain K12) GN=aceA PE=1 SV=1
☐ Check to include this hit in error tolerant search or archive report

| | Query | Observed | Mr(expt) | Mr(calc) | ppm | Miss | Score | Expect | Rank | Unique | Peptide |
|---|---|---|---|---|---|---|---|---|---|---|---|
| ☑ | 1 | 1495.9380 | 1494.9307 | 1494.8770 | 35.9 | 0 | 64 | 1.5e-005 | 1 | U | R.LAADVTGVPTLLVAR.T |
| ☑ | 3 | 1749.9370 | 1748.9297 | 1748.8733 | 32.2 | 0 | 24 | 0.29 | 1 | U | R.WEGITRPYSAEDVVK.L |

**Proteins matching the same set of peptides:**
ACEA_ECOL6 **Mass:** 47782 **Score:** 87 **Matches:** 2(1) **Sequences:** 2(1)
Isocitrate lyase OS=Escherichia coli O6:H1 (strain CFT073 / ATCC 700928 / UPEC) GN=aceA PE=3 SV=1

---

**Peptide matches not assigned to protein hits:** (no details means no match)

| | Query | Observed | Mr(expt) | Mr(calc) | ppm | Miss | Score | Expect | Rank | Unique | Peptide |
|---|---|---|---|---|---|---|---|---|---|---|---|
| ☑ | 2 | 1727.9194 | 1726.9122 | 1726.8824 | 17.2 | 1 | 19 | 0.88 | 1 | | LGNGMEGEPWLKAAVR |
| ☑ | 4 | 2711.4304 | 2710.4231 | 2709.2817 | 421 | 2 | 15 | 1.8 | 1 | | FHGGAAKIMSGNSETETQEVGFKER |

---

## Search Parameters

| | | |
|---|---|---|
| **Type of search** | : | **MS/MS Ion Search** |
| **Enzyme** | : | **Trypsin** |
| **Fixed modifications** | : | **Carboxymethyl (C)** |
| **Variable modifications** | : | **Oxidation (M)** |
| **Mass values** | : | **Monoisotopic** |

```
Protein Mass           : Unrestricted
Peptide Mass Tolerance : ± 100 ppm (# 13C = 1)
Fragment Mass Tolerance: ± 1 Da
Max Missed Cleavages   : 2
Instrument type        : MALDI-TOF-TOF
Number of queries      : 4
```

| **Mascot:** http://www.matrixscience.com/ |
|---|
