# Supplementary material for: How Bacteria Change after Exposure to Silver Nanoformulations: Analysis of the Genome and Outer Membrane Proteome
Source: Pathogens. 2021 Jun 29;10(7):817. doi: 10.3390/pathogens10070817 (PMC8308822; doi:10.3390/pathogens10070817)
Supplement: Supplementary file 1 [file pathogens-10-00817-s001.zip › supplementary file S1.zip!/Raporty (pod publikacje)/Reports (BW25113)/Spot no. 7.pdf]

Mascot Search Results

```
User              : sabina
Email             :
Search title      : MS_33175_281474976711919
Database          : SwissProt 2012_06 (536489 sequences; 190389898 residues)
Taxonomy          : Escherichia coli (22924 sequences)
Timestamp         : 18 Feb 2019 at 13:01:20 GMT
Top Score         : 87 for DGAL_ECOL6, D-galactose-binding periplasmic protein OS=Escherichia coli O6:H1 (strain CFT073 / ATCC 700928 / UPEC) G
```

Mascot Score Histogram

Protein score is -10*Log(P), where P is the probability that the observed match is a random event.
Protein scores greater than 56 are significant (p<0.05).

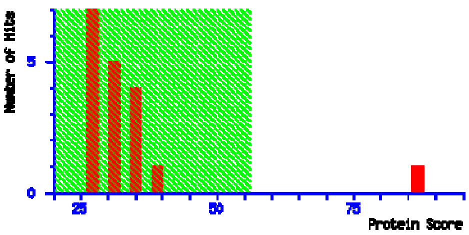

Concise Protein Summary Report

Format As | Concise Protein Summary | Help

Significance threshold p< 0.05 Max. number of hits AUTO

Re-Search All | Search Unmatched

```
1.   DGAL_ECOL6   Mass: 35690   Score: 87   Expect: 4.8e-005  Matches: 13
     D-galactose-binding periplasmic protein OS=Escherichia coli O6:H1 (strain CFT073 / ATCC 700928 / UPEC) GN=mglB PE=3 SV=1
     DGAL_ECOLI   Mass: 35690   Score: 87   Expect: 4.8e-005  Matches: 13
     D-galactose-binding periplasmic protein OS=Escherichia coli (strain K12) GN=mglB PE=1 SV=1
```

```
2.   DNLJ_ECO57   Mass: 73617   Score: 41   Expect: 2  Matches: 11
     DNA ligase OS=Escherichia coli O157:H7 GN=ligA PE=3 SV=3
     DNLJ_ECO5E   Mass: 73617   Score: 41   Expect: 2  Matches: 11
     DNA ligase OS=Escherichia coli O157:H7 (strain EC4115 / EHEC) GN=ligA PE=3 SV=1
     DNLJ_ECOLU   Mass: 73589   Score: 34   Expect: 9.6  Matches: 10
     DNA ligase OS=Escherichia coli O17:K52:H18 (strain UMN026 / ExPEC) GN=ligA PE=3 SV=1
     DNLJ_ECOHS   Mass: 73533   Score: 34   Expect: 9.8  Matches: 10
     DNA ligase OS=Escherichia coli O9:H4 (strain HS) GN=ligA PE=3 SV=1
     DNLJ_ECO24   Mass: 73618   Score: 34   Expect: 10  Matches: 10
     DNA ligase OS=Escherichia coli O139:H28 (strain E24377A / ETEC) GN=ligA PE=3 SV=1
     DNLJ_ECO55   Mass: 73618   Score: 34   Expect: 10  Matches: 10
     DNA ligase OS=Escherichia coli (strain 55989 / EAEC) GN=ligA PE=3 SV=1
     DNLJ_ECO8A   Mass: 73618   Score: 34   Expect: 10  Matches: 10
     DNA ligase OS=Escherichia coli O8 (strain IAI1) GN=ligA PE=3 SV=1
     DNLJ_ECOSE   Mass: 73618   Score: 34   Expect: 10  Matches: 10
     DNA ligase OS=Escherichia coli (strain SE11) GN=ligA PE=3 SV=1
     DNLJ_ECO7I   Mass: 73533   Score: 33   Expect: 12  Matches: 10
     DNA ligase OS=Escherichia coli O7:K1 (strain IAI39 / ExPEC) GN=ligA PE=3 SV=1
```

Search Parameters

```
Type of search          : Peptide Mass Fingerprint
Enzyme                  : Trypsin
Variable modifications  : Carbamidomethyl (C),Oxidation (M)
Mass values             : Monoisotopic
Protein Mass            : Unrestricted
Peptide Mass Tolerance  : ± 100 ppm
Peptide Charge State    : 1+
Max Missed Cleavages    : 1
Number of queries       : 63
```

Mascot: http://www.matrixscience.com/
