# Supplementary material for: How Bacteria Change after Exposure to Silver Nanoformulations: Analysis of the Genome and Outer Membrane Proteome
Source: Pathogens. 2021 Jun 29;10(7):817. doi: 10.3390/pathogens10070817 (PMC8308822; doi:10.3390/pathogens10070817)
Supplement: Supplementary file 1 [file pathogens-10-00817-s001.zip › supplementary file S1.zip!/Raporty (pod publikacje)/Reports (BW25113)/Spot no. 8.pdf]

Mascot Search Results

```
User            : sabina
Email           :
Search title    : E. coli_trypsin_100ppm
MS data file    : 281474976711946.mgf
Database        : SwissProt 2012_06 (536489 sequences; 190389898 residues)
Taxonomy        : Escherichia coli (22924 sequences)
Timestamp       : 19 Feb 2019 at 10:57:10 GMT
Protein hits    : SODF_ECOLI Superoxide dismutase [Fe] OS=Escherichia coli (strain K12) GN=sodB PE=1 SV=2
```

Mascot Score Histogram

Ions score is -10*Log(P), where P is the probability that the observed match is a random event.
Individual ions scores > 29 indicate identity or extensive homology (p<0.05).
Protein scores are derived from ions scores as a non-probabilistic basis for ranking protein hits.

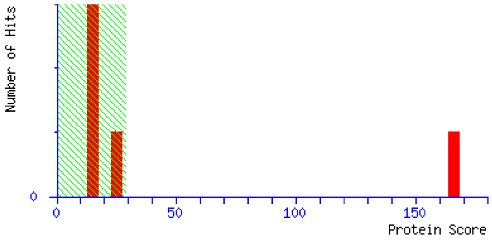

Peptide Summary Report

Format As | Peptide Summary | Help

Significance threshold p< 0.05 | Max. number of hits AUTO

Standard scoring ◉ MudPIT scoring ○ | Ions score or expect cut-off 15 | Show sub-sets 0

Show pop-ups ◉ Suppress pop-ups ○ | Sort unassigned Decreasing Score | Require bold red ☐

Select All | Select None | Search Selected | ☐ **Error tolerant** | Archive Report

1. SODF_ECOLI **Mass:** 21311 **Score:** 166 **Matches:** 2(2) **Sequences:** 2(2)
Superoxide dismutase [Fe] OS=Escherichia coli (strain K12) GN=sodB PE=1 SV=2
☐ Check to include this hit in error tolerant search or archive report

	Query	Observed	Mr(expt)	Mr(calc)	ppm	Miss	Score	Expect	Rank	Unique	Peptide
☑	1	2547.4268	2546.4195	2546.2859	52.5	0	111	4.6e-010	1	U	R.NARPGYLEHFWALVNWEFVAK.N
☑	3	3867.1226	3866.1153	3865.9414	45.0	0	55	0.00012	1	U	K.LAIVSTSNAGTPLTTDATPLLTVDVWEHAYYIDYR.N

Proteins matching the same set of peptides:
SODF_ECOL6 **Mass:** 21311 **Score:** 166 **Matches:** 2(2) **Sequences:** 2(2)
Superoxide dismutase [Fe] OS=Escherichia coli O6:H1 (strain CFT073 / ATCC 700928 / UPEC) GN=sodB PE=3 SV=2
SODF_ECO57 **Mass:** 21311 **Score:** 166 **Matches:** 2(2) **Sequences:** 2(2)
Superoxide dismutase [Fe] OS=Escherichia coli O157:H7 GN=sodB PE=3 SV=2

Peptide matches not assigned to protein hits: (no details means no match)

	Query	Observed	Mr(expt)	Mr(calc)	ppm	Miss	Score	Expect	Rank	Unique	Peptide
☑	2	3680.8701	3679.8628	3679.7617	27.5	1	16	0.73	1		DTGHFGMIDAIDDPQVFAALFGAAEAWLKSQGASK + Oxidation (M)

Search Parameters

```
Type of search          : MS/MS Ion Search
Enzyme                  : Trypsin
Fixed modifications     : Carboxymethyl (C)
Variable modifications  : Oxidation (M)
Mass values             : Monoisotopic
Protein Mass            : Unrestricted
Peptide Mass Tolerance  : ± 100 ppm (# 13C = 1)
Fragment Mass Tolerance : ± 1 Da
Max Missed Cleavages    : 2
Instrument type         : MALDI-TOF-TOF
Number of queries       : 3
```

Mascot: http://www.matrixscience.com/
