# Supplementary material for: How Bacteria Change after Exposure to Silver Nanoformulations: Analysis of the Genome and Outer Membrane Proteome
Source: Pathogens. 2021 Jun 29;10(7):817. doi: 10.3390/pathogens10070817 (PMC8308822; doi:10.3390/pathogens10070817)
Supplement: Supplementary file 1 [file pathogens-10-00817-s001.zip › supplementary file S1.zip!/Raporty (pod publikacje)/Reports (BW25113)/Spot no. 9.pdf]

Mascot Search Results

User : sabina
Email :
Search title : MS_33343_281474976711926
Database : SwissProt 2012_06 (536489 sequences; 190389898 residues)
Taxonomy : Escherichia coli (22924 sequences)
Timestamp : 18 Feb 2019 at 13:21:44 GMT
Top Score : 128 for YAHK_ECOLI, Uncharacterized zinc-type alcohol dehydrogenase-like protein YahK OS=Escherichia coli (strain K12) GN=y

Mascot Score Histogram

Protein score is -10*Log(P), where P is the probability that the observed match is a random event.
Protein scores greater than 56 are significant ($p<0.05$).

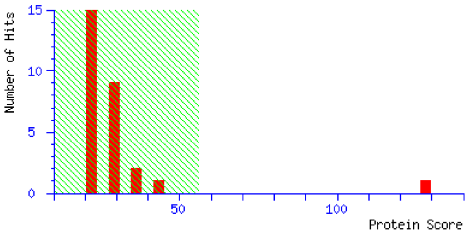

Concise Protein Summary Report

Format As | Concise Protein Summary | Help

Significance threshold p< 0.05 Max. number of hits AUTO

Re-Search All | Search Unmatched

1. YAHK_ECOLI **Mass:** 37954 **Score:** 128 **Expect:** 3.6e-009 **Matches:** 15
Uncharacterized zinc-type alcohol dehydrogenase-like protein YahK OS=Escherichia coli (strain K12) GN=yahK PE=1 SV=1
PAAF_ECOLI **Mass:** 27220 **Score:** 23 **Expect:** 1.1e+002 **Matches:** 4
2,3-dehydroadipyl-CoA hydratase OS=Escherichia coli (strain K12) GN=paaF PE=1 SV=1

---

2. RPI_ECOLX **Mass:** 15746 **Score:** 46 **Expect:** 0.62 **Matches:** 7
Protein D OS=Escherichia coli GN=D PE=4 SV=1

Search Parameters

Type of search : Peptide Mass Fingerprint
Enzyme : Trypsin
Variable modifications : Carbamidomethyl (C),Oxidation (M)
Mass values : Monoisotopic
Protein Mass : Unrestricted
Peptide Mass Tolerance : ± 100 ppm
Peptide Charge State : 1+
Max Missed Cleavages : 1
Number of queries : 45

Mascot: http://www.matrixscience.com/
