# Supplementary material for: How Bacteria Change after Exposure to Silver Nanoformulations: Analysis of the Genome and Outer Membrane Proteome
Source: Pathogens. 2021 Jun 29;10(7):817. doi: 10.3390/pathogens10070817 (PMC8308822; doi:10.3390/pathogens10070817)
Supplement: Supplementary file 1 [file pathogens-10-00817-s001.zip › supplementary file S1.zip!/Raporty (pod publikacje)/Reports (BW25113)/Spot no.1.pdf]

{MATRIX SCIENCE} Mascot Search Results

User : sabina
Email :
Search title : E. coli_trypsin_100ppm
MS data file : 281474976711954.mgf
Database : SwissProt 2012_06 (536489 sequences; 190389898 residues)
Taxonomy : Escherichia coli (22924 sequences)
Timestamp : 19 Feb 2019 at 12:14:53 GMT
Protein hits : FLIC_ECOLI Flagellin OS=Escherichia coli (strain K12) GN=fliC PE=1 SV=2

Mascot Score Histogram

Ions score is -10*Log(P), where P is the probability that the observed match is a random event.
Individual ions scores > 30 indicate identity or extensive homology (p<0.05).
Protein scores are derived from ions scores as a non-probabilistic basis for ranking protein hits.

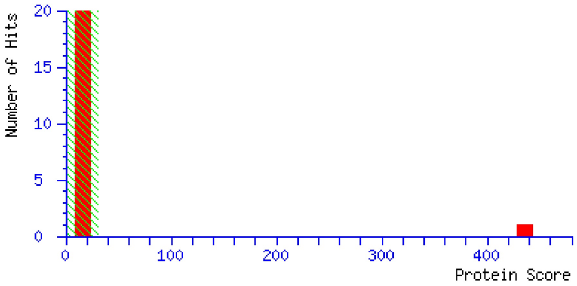

Peptide Summary Report

Format As | Peptide Summary | Help

Significance threshold p< 0.05 | Max. number of hits AUTO

Standard scoring ◉ MudPIT scoring ○ | Ions score or expect cut-off 15 | Show sub-sets 0

Show pop-ups ◉ Suppress pop-ups ○ | Sort unassigned Decreasing Score | Require bold red ☐

Select All | Select None | Search Selected | ☐ **Error tolerant** | Archive Report

1. FLIC_ECOLI **Mass:** 51265 **Score:** 436 **Matches:** 3(3) **Sequences:** 3(3)
Flagellin OS=Escherichia coli (strain K12) GN=fliC PE=1 SV=2
☐ Check to include this hit in error tolerant search or archive report

	Query	Observed	Mr(expt)	Mr(calc)	ppm	Miss	Score	Expect	Rank	Unique	Peptide
☑	1	1420.8625	1419.8553	1419.7358	84.2	1	109	7.5e-010	1	U	K.ALDDAIASVDKFR.S
☑	3	2076.2373	2075.2300	2075.0899	67.5	2	121	3.6e-011	1	U	K.TTDPLKALDDAIASVDKFR.S
☑	4	2629.4749	2628.4676	2628.2739	73.7	0	206	1.3e-019	1	U	R.NANDGISVAQTTEGALSEINNNLQR.V

Peptide matches not assigned to protein hits: (no details means no match)

	Query	Observed	Mr(expt)	Mr(calc)	ppm	Miss	Score	Expect	Rank	Unique	Peptide
☑	2	1516.9398	1515.9325	1514.7875	756	1	19	0.55	1		GRVGPGELMVIDTR + Oxidation (M)

Search Parameters

Type of search : MS/MS Ion Search
Enzyme : Trypsin
Fixed modifications : Carboxymethyl (C)
Variable modifications : Oxidation (M)
Mass values : Monoisotopic
Protein Mass : Unrestricted
Peptide Mass Tolerance : ± 100 ppm (# ^{13}C = 1)
Fragment Mass Tolerance: ± 1 Da
Max Missed Cleavages : 2
Instrument type : MALDI-TOF-TOF
Number of queries : 4
